# Polyphenols in Ruminant Nutrition and Their Effects on Reproduction

**DOI:** 10.3390/antiox11050970

**Published:** 2022-05-14

**Authors:** Drago Bešlo, Gloria Došlić, Dejan Agić, Vesna Rastija, Marcela Šperanda, Vesna Gantner, Bono Lučić

**Affiliations:** 1 Faculty of Agrobiotechnical Sciences Osijek, University J. J. Strossmayer Osijek, Vladimira Preloga 1, HR-31000 Osijek, Croatia; gloriadoslic@gmail.com (G.D.); dagic@fazos.hr (D.A.); vrastija@fazos.hr (V.R.); msperanda@fazos.hr (M.Š.); vgantner@fazos.hr (V.G.); 2 Ruđer Bošković Institute, NMR Centre, Bijenička cesta 54, HR-10000 Zagreb, Croatia

**Keywords:** plant polyphenols, reproduction, antioxidant activity, farm animals, reactive oxygen species, reactive nitrogen species, free radicals

## Abstract

The feeding of domestic animals with diets in which polyphenols are present is increasingly attracting the attention of nutritionists and scientists. This review summarizes the knowledge regarding polyphenols’ possible positive and negative effects and their bioavailability. The bioavailability of substances is a prerequisite for any postabsorption effect in vivo. Positive and negative properties have been confirmed in previous studies on the diets of domestic animals rich in polyphenols, such as secondary metabolites of plants. Free radicals are formed in every organism, leading to oxidative stress. Free radicals are highly reactive molecules and can react in cells with macromolecules and can cause damage, including in reproductive cells. Some polyphenols at specific concentrations have antioxidant properties that positively affect animal reproduction by improving the quality of male and female gametes. The intake of phytoestrogens that mimic estrogen function can induce various pathological conditions in the female reproductive tract, including ovarian, fallopian, and uterine dysfunction. The metabolism of genistein and daidzein yields the metabolites equol and p-phenyl-phenol, leading to a decline in cow fertilization. The findings so far confirm that numerous questions still need to be answered. This review points out the importance of using polyphenols that have both benificial and some unfavorable properties in specific diets.

## 1. Introduction

Free radical oxygen molecules are formed during aerobic cellular metabolism, containing one or more unpaired electrons. Free radicals can bind to various molecules and damage membranes, nucleic acids, and proteins [1]. In recent years, the plants used in feed have served as sources of different bioactive compounds for animals [2]. In addition, nutrient compounds play a very important role in protecting against the effects of free radicals [3,4]. An antioxidant dietary supplement can improve an animal’s productive status by altering metabolic processes. ROS (reactive oxygen species) production is a common physiological process in various organs, including the testes, which can lead to male infertility, although antioxidants can help [5,6]. It has been reported that antioxidants might be beneficial against the detrimental effects of leukocyte-derived ROS on sperm motility [7] (shown for vitamins C and E, dimethylsulfoxide, catalase, hypotaurine, N-acetylcysteine, and reduced glutathione) and function [8] (shown for ethylcysteine and vitamin E) due to the association of ROS overproduction with male infertility.

The reproductive rate significantly limits the livestock production efficiency. In the last two decades, there has been a growing interest in the use of herbal supplements in animal husbandry. Namely, the addition of herbs and their derivatives has improved animal health. This improvement is attributed to secondary plant metabolites or polyphenols. The findings to date confirm that polyphenols have antioxidant, immunomodulatory, antimutagenic, and anti-inflammatory effects. By restricting the use of antibiotics in livestock production, the use of plant polyphenols is increasingly being resorted to. The low intestinal absorption and low concentration in the target cells reduce their antioxidant effect. In this review, we will look at previous research on the bioavailability of polyphenols in reproductive organs [9].

There is evidence of negative effects of feed with high levels of phytochemicals and polyphenols on animal homeostasis, especially on sheep reproduction [10] associated with a high rate of infertility, affecting embryo survival and fetal development. In 1982–1983, an analogous result, i.e., serious fertility disturbances indicating estrogenic stimulation, was obtained after feeding cows with red clover plants rich in isoflavones, a subclass of polyphenols [11]. Similar results were also reported in other studies in which domestic animals were fed with soybean or linseed [10].

There are few studies that provide detailed measurements of the effects of foods rich in polyphenols that can act as phytoestrogens on the reproduction of domestic animals. In the study by Mustonen et al. [12], two groups of sheep of equal size were monitored in an experiment. In another study [13], the effects of condensed tannins and saponin in animal fed supplementation on reproductive performance in Barki ewes were analyzed. The question of the presence, diversity and optimal concentration of polyphenols in traditional ruminant nutrition is raised. The answer to this question was given in the study by Fraisse et al. [14], who analyzed the composition of chemicals in mountain pasture grass. This favorite nutrition of ruminants is genetically and epigenetically selected or optimized, together with rumen microflora, which helps and directs the digestion and absorption of nutrients. The presence of phytoestrogens in the milk of cows fed a polyphenol-rich diet was also studied [15], and it was found that their concentration was much higher when the animals were fed by legumes.

Immune dysfunction is caused by various factors, including changes in relevant immune regulators and environmental stress, and nutrition may play an essential role in immunity by interfering with proinflammatory cytokine synthesis, immune cell regulation, and gene expression [16]. Polyphenols can promote immunity to foreign pathogens via various pathways. Namely, different immune cells can express multiple types of receptors that recognize and allow the cellular uptake of polyphenols, thereby activating signaling pathways and initiating immune responses [16]. Furthermore, the polyphenols can induce epigenetic changes in cells, and they can be used to regulate intestinal mucosal immune responses, allergic diseases, and antitumor immunity.

Indeed, the balance between the harms and benefits of the use of polyphenols could argue for the use of polyphenols in animals used for meat production, as possible negative effects disappear at the end of the animal’s production cycle [10]. The reproductive outcome may also be affected by improper polyphenol intake. Incorrect ingestion can affect offspring (due to changes affecting gene expression or programming) and the future health of offspring. These genetic changes can result not only from parenteral exposure but also from the use of assisted reproductive techniques (ART) [10]. Genetic changes can be observed during parental exposure, as well as with the use of ART. Polyphenols are used as antioxidants and antibacterial compounds in ART, which certainly includes protection in gamete or embryo breeding. The simultaneous addition of green tea polyphenol, IGF-1, and glucose to cattle in a maturation medium increased the intracellular glutathione concentration in oocytes after in vitro maturation and improved the embryonic development and blastocyst quality [17].

The positive characteristics of polyphenol applications in vivo and in vitro should be further investigated before they are systematically used in practice as supplements to the basic livestock diet.

The aim of this article is to review the literature on the current state of knowledge in the use of polyphenols in animal nutrition, as well as proposals for future research giving importance to the formation of a proper diet and the introduction of new plant species or materials and dietary supplements for adequate access to reproductive functions and phases.

## 2. Polyphenols and Their Benefits

“Let food be your medicine, and medicine your food”, said Hippocrates more than 2000 years ago, showing that the benefits of natural sources of healthy food have been appreciated since ancient times [18]. Plant foods, including fruits and vegetables containing active substances, play a key role in human and animal health. Plants synthesize polyphenols under stressful conditions during adaptation to their environment. Polyphenols are an important source of active substances in pharmaceutical products [19]. The widespread use of polyphenols as secondary metabolites is an essential part of animal and human nutrition and is of great interest to scientists because of their biological properties. In the last few decades, scientists have been paying close attention to the health benefits of polyphenols [20,21]. Although the beneficial effects of polyphenols in both humans and animals have been confirmed, there are concerns regarding the potential health hazards of excessive polyphenol consumption [22,23]. The most vulnerable groups are pregnant animals and their fetuses [24]. Therefore, it is essential to understand the impacts of plant polyphenol consumption on reproductive health.

In plants, these compounds are usually synthesized as defenses against physiological and environmental stimuli. More attention has been paid in recent years to the benefits of polyphenols to human and animal health. This has been observed based on the chemical and biological activity and the obtained results [25,26,27]. Polyphenols have an advantage over other substances due to their good availability, low toxicity, and specific activity, while their biggest disadvantage is their fast metabolism and low bioavailability. A complex mixture of polyphenols is found in food, and various factors affect their diversity, primarily environmental (e.g., rain, pedological soil composition, sun exposure) and biochemical (e.g., storage conditions, degree of maturity, and method of preparation) factors [9]. Glycosides and aglycones of polyphenols are the most important plant secondary metabolites in both human and animal nutrition, having significant health effects [28,29]. A review of the literature on polyphenols, which includes more than 20,000 papers, confirmed that a significant proportion of these molecules have inhibitory activity against enzymes, as well as antitumor, anti-inflammatory, antibacterial, and antifungal activities [30,31], reflecting the extensive benefits of polyphenols for the animal community, including improvements in memory and cognition in animals and humans [32]. The bioavailability and kinetics of different polyphenols are very variable, so the knowledge of the fate of these compounds is quite unclear. In addition, based on their intensive metabolism in the gut and liver [33,34] of the parent compound, circulating metabolites very often differ from the parent compound, which further complicates the study of in vitro biological activity in animal models. From the above, it can be concluded that understanding the kinetics and bioavailability of polyphenols is crucial to know and understand the health benefits of these compounds.

## 3. Division of Polyphenols and Their Sources in the Diet

The term “polyphenol” is used for compounds synthesized exclusively by the shikimin–phenylpropanoid and shikimin–polyketide pathways, which are constructed of more than one phenolic moiety and do not exhibit fundamental nitrogen functionality [35]. Polyphenols are plants or synthetic compounds consisting of one or more phenolic units. Most of them are glycosylated or can bind to other phenols. In addition, they can conjugate with other compounds such as glucuronic acid, galacturonic acid, or glutathione during metabolism [36]. The diversity and wide distribution of polyphenols in plants have led to different methods of categorizing these natural compounds [35,36]. Polyphenols are classified according to their source of origin, biological function, and chemical structure. In addition, most polyphenols in plants are present in the form of glycosides with different carbohydrate units and acylated carbohydrates at different positions of the polyphenolic scaffold. The classification of polyphenols in this article is based on the chemical structure of the aglycone. Thus, polyphenols are divided into two main groups, namely flavonoids and non-flavonoids [35].

### 3.1. Flavonoids

**Figure 1:**
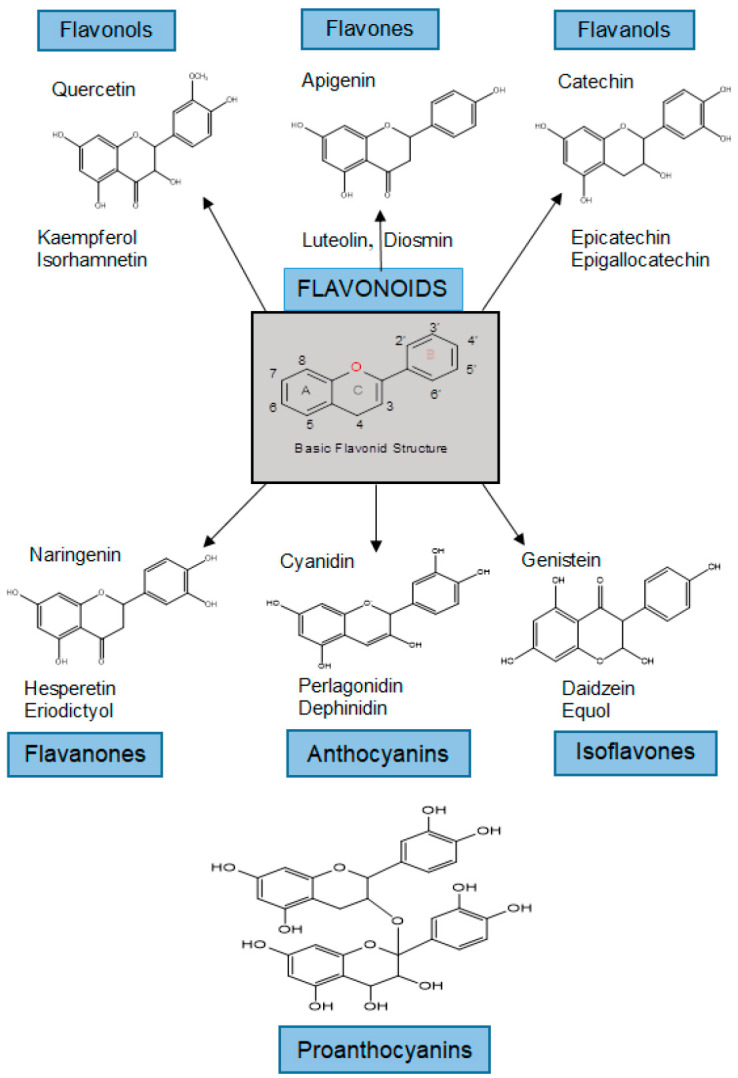
Classification and examples of structures of flavonoids.

The class of flavonoids contains more than 4000 low molecular weight secondary plant metabolites. They are formed from aromatic amino acids [36,37]. Flavonoids are classified into flavonols, flavones, flavanols, flavanones, anthocyanins, isoflavones, and proanthocyanidins (Figure 1) [37,38].

The basic part of the structure of flavonoids is the core, which consists of 15 carbon atoms arranged in three rings (C6-C3-C6) or a diphenylpropane skeleton, designated A, B, and C (Figure 1). Flavonoids are usually found as glycosylated derivatives in plants and contribute to the attractive colors of the flowers, leaves, and fruits [39]. The flavones apigenin and luteolin are commonly found in cereals and aromatic herbs (parsley, rosemary, and thyme), while their hydrogenated analogues hesperetin and naringin are found almost exclusively in citrus fruits. The flavonols quercetin and kaempferol are abundant in vegetable peels and fruits, with the exception of onions. Isoflavones are the most abundant in legumes, such as soybeans, black beans, and chickpeas. The flavanols catechin, epicatechin, epigallocatechin, and their gallate esters are ubiquitous in plants. Anthocyanidins and their glycosides (anthocyanins) are natural pigments and are most abundant in berries and red grapes. Flavonoids play various roles in the ecology of plants. Because of their attractive colors, flavones, flavonols, and anthocyanidins can serve as visual signals for pollinating insects. Because of their bitterness, catechins and other flavonols can provide a defense system against insects that are harmful to plants. They can also act as stress protectants in plant cells by trapping the ROS produced by the photosynthetic electron transport system. In addition, due to their favorable UV absorption, flavonoids protect plants from the sun’s UV radiation and remove the ROS produced by UV rays [39].

### 3.2. Non-Flavonoids

**Figure 2:**
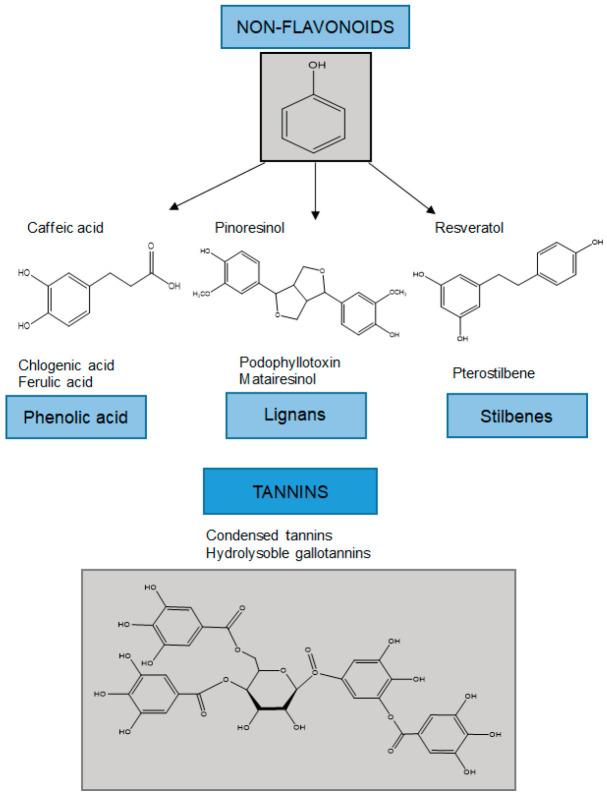
Classification of non-flavonoids and examples of their chemical structures.

Non-flavonoids can be classified into lower molecular weight compounds such as phenolic acids, lignans, and stilbenes, and more complex structures such as tannins (Figure 2).

The structural characteristics of simpler non-flavonoids are described below.

#### 3.2.1. Phenolic Acids

Phenolic acids are abundant in food and are divided into two classes: benzoic acid derivatives and cinnamic acid derivatives. Phenolic acids and flavonoids are the most abundant polyphenols in foods—they account for about one-third and two-thirds of the total sources, respectively [36,37].

The content of hydroxybenzoic acid in edible plants is generally low, except in some red fruits, black radish, and onions, which may have concentrations of several tens of mg/kg fresh mass. Hydroxycinnamic acids are more common than hydroxybenzoic acids and consist mainly of p-coumaric, caffeic, ferulic, synaptic, chlorogenic, and rosmarinic acids [38].

#### 3.2.2. Stilbenes

Stilbenes are a small and important class of non-flavonoid polyphenols characterized by a 14-carbon skeleton. They are built of two benzene rings connected by an ethylene bridge (Figure 1) [37]. In the central part of the structure, two aromatic rings are connected to ethene, and ethene hydrogen can be in the *cis* and *trans* positions. In nature, stilbenes occur most frequently in the form of *trans* stereoisomers. To date, more than 400 different stilbene compounds have been identified, most of which are derived from *trans* resveratrol (3,5,4′-trihydroxy-*trans* stilbene). Due to the complexity of qualitative–quantitative stilbene analysis, most studies have focused on simple stilbenes, such as resveratrol, piceid, pterostilbene, and piceatannol.

The knowledge on stilbenes mainly relates to the protection of plants against biotic (phytopathogenic microorganisms and herbivores) and abiotic (e.g., radiation and tropospheric ozone) stress. In this way, they repel attacks by having a direct toxic effect on the pathogen, while on the other hand they act as antioxidants and protect the cells from oxidative stress [37].

#### 3.2.3. Lignans

Lignans are diphenolic compounds that contain a 2,3-dibenzylbutane structure formed by the dimerization of two cinnamic acid residues (Figure 1). The richest source is flaxseed, which contains secoisolariciresinol and matairesinol. One of the most common forms of lignans is secoisolariciresinol (2-(4-hydroxy-3-methoxybenzyl)-3-(3-metoxybenzyl)butene-1-4-diol) diglycoside. Seicoisolariciresinol and matairesinol ingested with food are converted by the intestinal microflora into mammalian lignans, enterodiol and enterolactone, which are absorbed via the enterohepatic circulation. Mammalian lignans have a chemical structure similar to natural estrogen, and are thought to act as selective modulators of estrogen receptors and to have anticancer activity [40].

## 4. Appearance of Polyphenols

The distribution of polyphenols in plants at the tissue, cellular, and subcellular levels is not uniform. Insoluble polyphenols are found in the cell walls, while soluble polyphenols are found in the vacuoles of plant cells [41]. In most cases, foods contain complex mixtures of polyphenols. The outer layers of plants contain higher contents of polyphenols than the inner parts [38,42]. A variety of factors influence the polyphenol content in plants, including the degree of ripeness at harvest, environmental factors, processing, and storage. The degree of ripeness has a significant influence on the concentrations and proportions of the various polyphenols [34,38]. In general, it has been observed that the phenolic acid content decreases during ripening, while the anthocyanin concentration increases. They contribute to the healing of areas damaged by lignification, have antimicrobial properties, and their concentrations can increase after infection [43]. Plants synthesize polyphenols such as phenolic acids or flavonoids under optimal and suboptimal growth conditions, and they play key roles in developmental processes, mineralization, and reproduction. Under stressful conditions, their production is increased, helping plants to cope with environmental constraints [44] and improving the tolerance and adaptability of plants under suboptimal conditions [45].

Storage is another factor that can directly affect and change the polyphenol content due to slight oxidation [34,38]. A disproportionate polyphenol intake initially has no effect on the reproduction of the parents, but prolonged intake can cause epigenetic changes in the offspring that affect gene expression or programming and the future health or disease of the offspring [10]. The current knowledge suggests that feeding cows with red clover silage (*Tiofolium pratense*) has led to fertility disorders. The contents of estrogen and isoflavones in silage were found to be very high. After cessation of feeding, the disorders disappeared [11]. Mountain pastures known to have very different polyphenols should be used to assess the quality of animal feed during grazing [14]. Red clover has been confirmed to be the best source of phytoestrogens [15].

## 5. Absorption, Metabolism, and Elimination of Polyphenols

The intestinal absorption of polyphenols depends on their physicochemical properties, including molecular weight and extent of glycosylation and esterification. Polyphenols with higher molecular weights are less likely to be absorbed in the gut, as are anthocyanins, which carry a positive charge [36,46]. In general, polyphenols in the form of esters and glycosides are absorbed more slowly and less efficiently than aglycones (compounds that remain after the hydrolysis of phenolic glycosides and esters) and glucosides (glycosides derived from glucose) [36,37,46,47]. The reason for this is that glycosylated polyphenols are hydrophilic, so they cannot passively diffuse through the intestinal wall until they are hydrolysed [36,48,49,50]. After cleavage into the corresponding aglycones, polyphenols can be taken up in small (or large) intestinal enterocytes by passive diffusion or facilitated by active transport. Passive diffusion is probably the main absorption pathway for low-weight polyphenols such as phenolic acids or several flavonoid aglycones [51]. It has been demonstrated in vitro that glycosylated phenols can be transported through the intestinal cell wall in the jejunum of rats via active transport mechanisms [36,52]. Selective transporters are also thought to be involved in the uptake of polyphenols through the placenta [53,54], although the identity of these transporters is not yet clear. Polyphenols are extensively metabolized by phase I and II enzymes of xenobiotic metabolism as they pass through the small intestine and return to the liver after passing the first passage through the portal vein. Phase I reactions are mainly carried out by a subfamily of isoenzymes known as cytochrome P450-dependent mixed function oxidases (CYPs) [36]. They make the molecule more polar and are important in facilitating the phase II conjugation reactions that lead to excretion [53,54]. These reactions are very effective, suggesting the absence or low amounts of free aglycones during polyphenol metabolism [55]. The identification of conjugated metabolites has only been studied for a few polyphenols, and data on the types of metabolites circulating in human plasma are limited [56]. It is known that these metabolites do not circulate freely in the blood but mostly bind to plasma proteins, especially albumin [57], and that the binding affinity of these metabolites to albumin depends on their chemical structure [58,59]. However, the degree of binding to albumin and the effects on the binding rate and biological activity of the metabolites are unclear [33]. Phase I and II enzymes were identified in the placenta. These enzymes are well characterized because they participate in the detoxification of drugs; however, the in vivo interactions with polyphenols have not been confirmed [60,61]. Nevertheless, in vitro assays and in vivo non-placental studies have clearly demonstrated that polyphenols can have complex effects on drug metabolism by activating and inhibiting the activity of CYP and phase II enzymes [57,62,63]. Finally, the effects of polyphenols on placental drug metabolism may be similar, but should be studied directly.

After biotransformation in phases I and II, weakly conjugated polyphenols re-enter the circulation, while generally conjugated polyphenols are excreted via the bile and enter the colon. The microflora hydrolyses glycosides into aglycones, which are then metabolized to various aromatic acids that are well absorbed through the colon barrier [64,65]. Ongoing studies should focus on the identification and quantification of microbial metabolites in both humans and animals and explore differences in metabolism of various polyphenols, depending on the composition of their microflora and diet. This is of great importance for active metabolites, as they may have a specific physiological effect [66]. The identification of metabolites can be a useful biomarker for phenol intake and it can help to determine the biological activity of specific conjugates derived from polyphenols present in vivo. The excretion of individual polyphenols varies depending on the type of compound, as has been confirmed in animal studies [66]. Depending on their size and degree of conjugation, most dietary phenolic metabolites are rapidly excreted in the urine or bile [33]. In general, the concentration of polyphenols excreted in urine is proportional to the maximum concentration of metabolites in plasma. However, there are some exceptions, such as anthocyanins, where the percentage of urinary excretion relative to the plasma concentration is very low [54]. This may be explained by the increased bile secretion or extensive metabolism of currently unidentified metabolites or unstable compounds.

The metabolites excreted in the bile and intestinal lumen can also be hydrolysed by bacterial catalysis via β-glucuronidases, which are able to release free aglycones from conjugated metabolites. As a result, the aglycones can be absorbed in the small or large intestine and undergo enterohepatic recycling. In this way, the metabolism and the delay of the first passage do not lead to complete excretion of the substance, but considerably prolong the elimination half-life [67,68].

## 6. Bioavailability of Polyphenols in Ruminants

Several studies in the literature on the bioavailability of polyphenols in ruminants indicate that the digestive system of ruminants absorbs only small amounts of these compounds [68]. Quercetin aglycone administered intraduodenally to lactating dairy cows has been shown to be absorbed to a similar extent as in pigs [69,70].

The administration of isoflavones (e.g., genistein or daidzein) to dairy cows via soybean meal or red rape silage results in increased concentrations of these isoflavones in both blood and milk [71,72]. Feeding cows silage containing red clover was found to increase the flavonoid content in milk when red clover was added at low and high concentrations. It was observed that the content of non-flavonoids decreased compared to silage containing white clover [73].

The administration of four different polyphenol-rich plant extracts (rosemary, grape, citrus and marigold) via the rumen cannula to sheep increased the concentrations of various polyphenols, such as epicatechins, in plasma [74]. These studies showed that at least some of the isoflavones contained in the diet are protected from degradation in the rumen and are available in the small intestine—the site of polyphenol absorption. In addition, there are several studies showing that feeding heifers a diet enriched with polyphenols alters the levels of several parameters related to rumen fermentation (e.g., pH and concentration of fermentation products in rumen juice) and the bacterial community in the rumen [75,76].

Similarly, some studies have shown that feeding animals flavonoids from plant extracts increases the numbers of lactate-consuming and propionate-producing bacteria [74,75]. These studies suggest that the composition of the microbiota in the rumen of ruminants, similar to monogastric animals, is modulated by plant polyphenols. Flavonoid degradation in the rumen has negative effects on the bioavailability of some monomeric forms of flavonoids, although microbial activity provides the opportunity for the efficient utilization of the polymeric forms of flavonoids. Flavonoids are absorbed in the small intestine in both monogastrics and ruminants, but a monomeric form is required for absorption [77,78].

In contrast to the results in newborn calves, the relative bioavailability of total flavonols from rutin was almost 8-fold higher compared to quercetin aglycone in adult cows. The low systemic availability of quercetin aglycone is attributed to the rate and extent of its microbial degradation. In an in vitro study, 90% of quercetin aglycone was degraded within the first 5 h of incubation [79]. When the effects on the rumen were bypassed via the intraduodenal administration of quercetin aglycone and rutin, the flavonol concentration in plasma was significantly increased by quercetin aglycone, whereas rutin showed no effect [80,81].

As is common in the large intestine of monogastric animals, microbes in the rumen can cleave the glycosidic portion of polymeric flavonoids, making them suitable for intestinal absorption [79]. Intraduodenally administered polymeric or glycosylated forms of flavonoids such as rutin cannot be absorbed in the small intestine, whereas quercetin (aglycone) is a form that can be readily absorbed. This example shows how microbial metabolism in ruminants leads to the greater bioavailability of glycosylated forms compared to aglycone forms of flavonoids. Regarding orally administered quercetin aglycone, the appearance of 3,4-dihydroxyphenylacetic acid, phloroglucinol, and 4-methylcatechol, degradation products of the aglycone form of quercetin during in vitro incubation with rumen inoculum, shows that it is rapidly degraded by microbes [79,80,81,82].

Since some rumen microbes (*Butyrivibrio*) also use the flavonoid glycoside rutinose and can cleave the heterocyclic ring, rutin can also be completely degraded [83]. Other rumen microbes such as *Butyrivibrio, Butyrivibrio fibrisolvens,* and *Selenomonas ruminantium* only cleave glucorhamnoside and produce quercetin and rutinose. Although these microbes can ferment the sugars, they cannot degrade the heterocyclic ring of quercetin [77,84]. Moreover, in a fecal inoculum incubated with rutin, degradation products were detected that originate from the cleavage of the C-ring of quercetin [84]. However, quercetin is absorbed by rutin, as flavonol concentrations in plasma increase after intraruminal administration of rutin and the relative bioavailability of total flavonols from rutin (67.3%) was higher than from the quercetin aglycone (100%) [82]. The degradation products of quercetin, whether from quercetin aglycone or rutin, can be further metabolized to volatile fatty acids (VFA) [85]. However, no difference was found in the degradation of quercetin aglycone or rutin to VFA.

Studies in monogamous animals have clearly shown that the small intestine is the main site of absorption of monomeric flavonoids. The absorption of flavonoids released from polymeric flavonoids occurs in the distal small intestine and in the large intestine after cleavage of the glycosides by the enteric microflora [69,85]. In ruminants, it is not known whether flavonoids are absorbed in the rumen epithelium, but it has been observed that flavonoid concentrations in plasma increase after the intraruminal administration of quercetin (30–35 min) [82] or after the consumption of isoflavones (1 h) [85]. This suggests that at least some flavonoids are likely to be absorbed in the rumen, as the average residence time of food in the rumen is much longer than 1 h. It appears that the flavonoids originating in the rumen are probably absorbed in the small intestine, as the total concentration of flavonols in plasma increases after intraduodenal quercetin infusion [78].

Microbial hydrolysis of the glycosidic forms has also been shown to be effective in the addition of peel extracts and grape seeds, which are rich in proanthocyanidins [77]. The dominant monomeric forms of proanthocyanidins in peel and grape seed extracts are catechin and epicatechin. The administration of grape seed extract and grapes resulted in measurable plasma concentrations of five different phenolic compounds in ruminants, including epicatechin [79]. The relatively high concentration of epicatechin and other phenols is probably the result of the microbial cleavage of polymeric proanthocyanidins. The microbial effects on the major catechins in green tea extracts (GTEx; halocatechin, epigalocatechin, catechin, epicatechin, epigallocatechin gallate, epicatechin gallate) are extensive, with catechin being the only form detected (at low levels) in plasma following the intraruminal administration of GTEx in dairy cows [86].

Murita and Terao [87] have proposed a mechanism by which glycosylated quercetin can cleave glucose. When quercetin monoglucose is taken up by enterocytes, the sodium-dependent glucose transporter 1 is introduced. In the enterocyte cytosol, β-glucosidase can cleave the glucose, and the product is the quercetin aglycone. Also in enterocytes, the quercetin aglycones can react with UDP–glucosyltransferase or phenol–sulfotransferase before entering the bloodstream. In conclusion, the monomeric forms of flavonoids are the best protected from degradation in ruminants, and their bioavailability is increased.

## 7. Effects of Polyphenol as Phytoestrogens

### 7.1. Benefits of Polyphenols in Ruminants

Improved reproduction in these literature reviews is associated with the antioxidant effects of polyphenolic compounds and their ability to mitigate the detrimental effects of ROS. It has been confirmed that some polyphenols due to structural similarity have the ability to emit estrogens. They are named phytoestrogens, and they can negatively affect fertilization.

**Figure 3:**
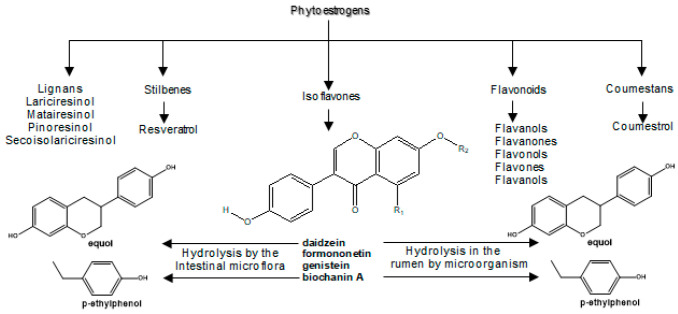
Classification of phytoestrogens and their metabolism.

Phytoestrogens form a group of compounds of plant origin that include isoflavones, flavonoids, coumestanes, stilbene, and lignans. Their biological transformations are illustrated in Figure 3.

As Wocławek-Potocka et al. [88] have shown, phytoestrogens are metabolized in the digestive system of mammals and ruminants. It has been experimentally observed that equol and p-ethylphenol are end products of phytoestrogen metabolism formed by hydrolysis with the help of microorganisms in the rumen. Thus, ruminants metabolize daidzein to equol and genistein to p-ethylphenol [88]. Isoflavones are found in high concentrations in soy products, while lignans are found in flax seeds, coumestans in clover, and stilbene in cocoa and grape products, especially in red wine and catechins in fruits [89,90,91].

**Figure 4:**
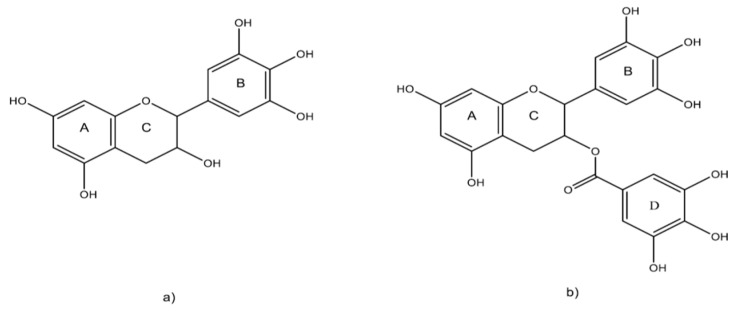
(**a**) Basic structure of catechin. (**b**) Chemical structure of EGCG.

The findings so far show that catechins can potentially improve reproductive health and represent an important area of research. Epigallocatechin-3-gallate (EGCG) is a bioactive compound that has the most promising antioxidant effect (Figure 4).

EGCG is a non-toxic antioxidant that can be used in the treatment of male infertility, oocyte fertilization rates, and embryo maturation rates [92]. Successful fertilization of oocytes depends on their maturation. Oocyte maturation in vitro is exposed to more oxygen than in the body [93,94]. Oxygen exposure allows the formation of oxygen-free radicals that can inhibit oocyte maturation [93,94]. The cause is probably that the concentration of glutathione in oocytes decreases after the formation of ROS [95,96,97].

**Figure 5:**
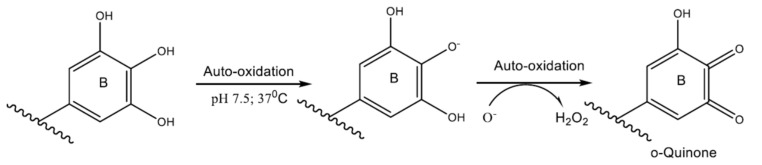
Superoxide-mediated chain reaction formation of o-quinone.

EGCG has a positive effect on infertility by protecting germ cells and eggs from damage [92]. EGCG primarily acts to regulate ROS by affecting the expression of catalase (CAT), superoxide dismutase 1 (SOD1), superoxide dismutase 2 (SOD2), and glutathione peroxide, positively affecting other enzymes and activity in germ cells and oocytes and actively altering antioxidant activity [90]. The oxidation of EGCG is associated with its autooxidation at pH 7.5 and 37 °C (Figure 5), leading to the formation of oxidative products such as Q-Quinone.

Scientists suggest that adequate intake of polyphenols [98], especially EGCG [99], in the daily diet can reduce the rate of sperm malformations and have a positive effect on the male and female reproductive systems in vitro [98,99]. Analogous results were obtained using antioxidants resveratrol and EGCG that prevented DNA damages in animal models (stallions) [100]. The efficiency and mechanism of action of polyphenols in vivo strongly depends on the extent of their biotransformation [101] in a living organism.

### 7.2. Adverse Effects of Phytoestrogenes in Ruminants

The assumption is that food from a natural source should be safe to consume. The most common antioxidants are polyphenols from plant foods [102], which has resulted in an increased interest in polyphenol intake from a variety of plant sources, including residues from plant processing. For these reasons, foods rich in polyphenols are recommended during conception and throughout pregnancy in order to supply the body with antioxidants in that state. Despite the beneficial effects, experimental studies have shown concern regarding the potentially dangerous overconsumption of polyphenols [12,23,24,25]. The most at-risk groups in this sense in animal husbandry are pregnant animals and their embryos, babies, and newborns. Several studies have been conducted to evaluate the adverse effects of isoflavones (Figure 3) on fertility. Isoflavones are phytoestrogens that are chemically similar to estradiol-17β and can bind to membranes and nuclear estrogen receptors, show estrogenic activity, and can alter reproductive function [103].

The most common source of exposure to phytoestrogens for ruminants is food obtained from soy, which is rich in the isoflavones genistein and daidzein, or from red clover containing 35.54% isoflavones (formononetin, biochanin A, daidzein, and genistein) and only 1.11% of other flavonoids [103,104]. These compounds are metabolized in the digestive tract into even stronger metabolites—*para*-ethyl-phenol and equol (Figure 3) [88]. In addition, the bioactivity of polyphenols depends on several factors, such as their molecular weight, conjugation with other derivatives, or the hydrolysis of enzymes in the digestive tract (stomach and intestines). The actions of enzymes from the digestive tract and microflora led to the formation of a new metabolite of polyphenols with different biological activity compared to those from the original compounds. For example, gastrointestinal bacteria can convert lignans (e.g., matairesinol, lariciresinol, and secoisolariciresinol; Figure 3) to more potent estrogenic “mammalian lignans” (enterodiol and enterolactone) [104]. Furthermore, feeding ruminants with the addition of catechins from green tea (GTE) intraruminally (orally) and intraduodenally showed that catechins were intensively metabolized by rumen microorganisms [105,106].

Red clover silage is rich in estrogen-emitting isoflavones, and it has been shown to cause fertility disorders in cows [11]. A frequently cited article related to the study of the negative effects of polyphenols as phytoestrogens is the work by Mustonen et al. [12], in which two equally large groups of sheep were observed. The first group was fed with a high concentration of phytoestrogens from red clover for five months before, during, and after the breeding season. The reproductive characteristics of the first group were compared with the control results of the grass-fed silage group [12]. In both groups, sheep fertility was not reduced, and the negative effect of phytoestrogens was attributed to increased fetal fluid volume, which can lead to a risk of premature vaginal prolapse.

Another study [13] analyzed the effects of condensed tannins and saponin as supplemented in animal feed given to Barki ewes on their reproduction [13]. The 30 ewes were classified into three equally sized experimental groups receiving different feed supplements, as well as another group (control) that did not receive supplements and received only basal nutrition. Animals in the three experimental groups received tannin extract (group 1), saponin extract (group 2), and both (group 3), respectively. No significant deviations were found in any of the 10 observed reproductive performance parameters, including serum cortisol and estradiol or progesterone concentrations. Compared to the control group, only statistically significant increases were observed in the serum insulin-like growth factor-1 (IGF-1) concentration in group 1 and serum insulin concentration in group 2. Additionally, significant changes in the concentrations of thyroid hormones 3 and 4 (T3 and T4) were also detected in some groups. It was concluded that the supplements applied to animal feed did not show a positive impact on reproductive performance in Barki ewes. Apparently, when breeding animals are fed plants rich in phytoestrogenic polyphenols from soybeans, flaxseed, clover, burdock seeds, rosemary leaves, or ground green tea leaves, extreme caution should be exercised [10].

In addition, the effects of clover phytoestrogen isoflavones on the hormonal balance and pregnancy of heifers were confirmed via the use of different concentrations of isoflavone aglycones (genistein, daidzein, biochanin A, and formononetin) [106]. Heifers were divided into two groups, where the first group was fed clover and the second corn silage for 20 weeks. Isoflavone concentrations were higher in the clover silage, resulting in hormonal imbalance and leading to the reduced fertility of clover-fed heifers during early pregnancy [106].

Phytoestrogens can disrupt reproductive processes in the targeted cells at different regulatory levels [88]. Mathieson and Kitts [106] studied the binding of phytoestrogens to the estradiol receptor in the pituitary and hypothalamus of ewes. Phytoestrogens can inhibit endogenous estrogen production in the ovaries, leading to disturbances in immune system regulation, follicle development, and estrus deficiency [107]. High concentrations of active phytoestrogen metabolites associated with lower P4 concentrations were found in corpus luteum (CL) tissues collected from soybean-fed heifers compared to animals fed a standard feed mixture [108]. CL produces P4, which is needed to establish and maintain pregnancy. Consequently, inhibiting P4 secretion by active phytoestrogen metabolites can disrupt CL function, thereby inducing a variety of disturbances during early pregnancy, including early embryonic mortality [88].

Phytoestrogens and their active metabolites (equol and para-ethyl-phenol) can inhibit LH-stimulated P4 secretion. Their adverse effect is indirect because it depends on the ability of phytoestrogens to inhibit LH- and PGE2-stimulated P4 production [108]. Feeding cows with foods high in soy may be the cause of estrus cycle disorders and several ovarian dysfunctions during early pregnancy [108].

Phytoestrogen metabolites (equal and p-ethyl-phenol) prevent the formation of bovine CL by preventing LH (luteinizing hormone) secretion (causing follicle and oocyte maturation, ovulation, and CL formation) and inhibiting progesterone P4 synthesis, which prepares the uterus for ovum implementation and pregnancy. Feeding cows with a strain that has a high concentration of phytosterols can disrupt the estrous cycle and cause ovarian dysfunction during early pregnancy, which it can lead to early embryonic death [107,108,109,110,111,112]. In a study where cows were fed with isoflavones in different concentrations (group I 1.0 ± 0.3 µmol/L, group II 4.0 ± 0.5 µmol/L, and group III 2.6 ± 0.9 µmol/L), the researchers came to the conclusion that the isoflavone concentration is a silent heat indicator in cows [113,114]. Isoflavones lead to reduced secretion of estradiol 17β in cows, resulting in a reduction in silent heat [115,116].

Therefore, different polyphenol affinities for the two ER subtypes (estrogen receptor) and different ER distributions in reproductive tissues have large impacts on the final outcome of polyphenol exposure [114,117]. In this way, polyphenols can intervene in the regulation of all reproductive processes through the hormonal modulation of neuro-hormones (gonadotropin-releasing hormone (GnRH) and oxytocin), gonadotropins, LH and follicle-stimulating hormone (FSH), steroids (E2, P4), and prostaglandins [10,118].

The results to date support the protective role of polyphenolic compounds—depending on the type of compound—under stress conditions, when harmful pathways such as oxidative stress and inflammatory processes are activated [10]. This suggests that polyphenolic compounds may lead to improved reproduction in stressed animals [118]. Several negative consequences of polyphenolic compounds in the reproduction of animals have been detected, as described above. However, it should be noted that diet is not a free choice in pharm animals, nor is the rumen microflora optimized to digest large amounts of polyphenolic compounds. There is insufficient information on the direct effects of different polyphenols on reproduction. Further studies are needed on the polyphenolic profiles of individual plants to confirm or refute these assumptions [119].

## 8. Antioxidant and Prooxidative Actions of Polyphenols

Free radicals are constantly generated in vivo both by accidents of chemistry and for specific metabolic purposes. When an imbalance between free radical generation and body defense mechanisms occurs, oxidative damage will spread over the cell targets (DNA, lipids, proteins) [19]. Therefore, this section will describe the positive antioxidant and also possible negative prooxidative effects of polyphenols in the diet of ruminants.

### 8.1. Antioxidant Properties of Polyphenols

ROS (reactive oxygen species) and RNS (reactive nitrogen species) play a dual role in the biological system, as they can be either harmful or beneficial to living cells [120]. The beneficial effects of ROS include physiological roles in cellular responses such as defense against infectious agents and in the functions of a number of cellular signaling systems [118]. Another useful example of low ROS concentrations is the triggering of a mitogenic reaction. In contrast, high concentrations of ROS can be important mediators of damage to cellular structures, including lipids and membranes, proteins, and nucleic acids (so-called oxidative stress (OxSt) [119,121]. The harmful effects of ROS are counterbalanced by the antioxidant effects of non-enzymatic antioxidants and antioxidant enzymes [114]. At the normal physiological level, ROS regulates the intracellular signaling cascade mediated by physiological mechanisms such as sperm fertilization and maturation response [114,122]. High levels favor the pathological condition. The treatment strategy should be aimed at lowering ROS [123].

Antioxidant mechanisms exist in all organisms that enable them to cope with the oxidative environment and help cells repair the damage caused by ROS [118]. These mechanisms can be divided into non-enzymatic and enzymatic. Antioxidant enzymes include CAT, SOD, GPx, glutathione reductase (GR), and GPx. SOD, CAT, and GPx are the three most abundant antioxidant enzymes, playing a key role in the removal of harmful oxygen products [13]. Glutathione (GSH) is considered an important representative of the non-enzymatic antioxidants present in oocytes and embryos [123,124,125]; other types of non-enzymatic antioxidants include vitamin C, vitamin E, selenium, Zn, carotene, and β-carotene. This balance is essential for the survival of organisms and their health [114].

ROS sustainability, sperm motility and fertilization potential disrupt OxSt at reproductive age, as shown by the presence of significantly higher ROS levels in the sperm of infertile men compared to fertile controls. The use of antioxidants from exogenous plants can probably improve male health. In OxSt, reproduction and fertility can be impaired, including impaired ovarian function, ovarian reduction, embryonic developmental disorders, and infertility [126,127]. Antioxidants are, therefore, key to maintaining redox balance in the ovaries to ensure normal ovarian function. However, their exact molecular mechanisms and roles are not yet fully understood.

Polyphenols are powerful antioxidants that complement and enhance the functions of antioxidant vitamins and enzymes to protect against oxidative stress caused by excess ROS. Although most of the evidence for the antioxidant activity of polyphenols is based on in vitro studies, there is growing evidence that they may act beyond antioxidant functions in vivo [35]. The modulation of cell signaling pathways by polyphenols could contribute significantly help to explaining the mechanisms of action of a polyphenol-rich diet [35,128,129].

The antioxidants we find in food can help by acting directly on ROS or by stimulating the endogenous defense system [130]. The property that most describes flavonoids is their capacity to protect the body from free radicals [131,132]. Flavonoids can prevent free radical damage in a variety of ways, one of which is the scavenging of various oxidizing types, i.e., superoxide anions (O_2_^•−^), hydroxyl radicals, or peroxy radicals. One method is direct scavenging, in which flavonoids are oxidized by radicals (R^•^), leading to the formation of less reactive and more stable molecules, which occurs via the following mechanism [19]:

flavonoid (OH) + R^•^ → flavonoid (O^•^) + RH

The flavonoid radical thus formed is stabilized by resonance. The unpaired electron becomes delocalized throughout the aromatic ring. The newly formed radical can go into further dimerization, dismutation, and recombination reactions with other radicals or oxides of quinone. The flavonoid radicals can react with oxygen to form quinone and superoxide anions. This reaction is responsible for the unwanted prooxidative effects of flavonoids. Quinone can be obtained via the reaction of a flavonoid radical with another radical [133]. Iron (Fe^2+^) and copper (Cu^+^) ions are essential for certain physiological functions of the cell, and as components of hemoproteins or as cofactors of various enzymes they can be involved in the antioxidant protection of the cell. Iron and copper ions are also responsible for the synthesis of hydroxyl radicals.

H_2_O_2_ + Fe^2+^ (Cu^+^) → ^•^OH + OH^-^ + Fe^3+^ (Cu^+^)

Flavonoids have the ability to chelate metal ions and form stable complexes with metal ions of the transition group (Fe^3+^, Al^3+^, Zn^2+^). The stoichiometry of the complex and the site of chelation depend on the nature of the flavonoid and on the pH [129,134].

### 8.2. Polyphenols as Prooxidans

Polyphenol compounds, including when present in low or moderate levels in animal nutrition, have positive effects on productive performance and health [119,132]. Among the various flavonoids, the flavonol quercetin is one of the most researched polyphenols, showing various health-promoting properties (antioxidant, anti-inflammatory, etc.) [78,133].

The content of quercetin in conventional ruminant food has not been extensively investigated so far. Several available studies have indicated the amounts of total polyphenols in grass (35.3 g/kg DM and maize silage (3.2 g/kg DM)) for cows grazing on permanent pasture, with polyphenol concentrations ranging from 19 to 32 g/kg [14,78,133]. In ruminants, quercetin undergoes intensive microbial fermentation in the stomach. According to previous research, compounds of 3,4-dihydroxyphenylacetic acid (3,4-DHPAA), phloroglucinol (PG), and some minor metabolites have been identified in humans, cows, and pigs [78,133].

Among other functions, free radicals are involved in energy production, cell growth regulation, and intercellular signaling. However, when there is an imbalance between the formation of free radicals and their neuralization, free radicals can react with lipids in the cell membrane, proteins, and enzymes and with DNA in the cell [132,134]. Under normal conditions, physiologically important intracellular levels of reactive oxygen species are maintained at low levels by various enzyme systems involved in vivo redox homeostasis [135,136]. Therefore, oxidative stress can also be viewed as an imbalance between prooxidants and antioxidants in the body. Free radicals play an irreplaceable role in phagocytosis as one of the important microbicidal systems [133,134], as well as in several biochemical reactions, for example, hydroxylation, carboxylation, peroxidation, and ribonucleotide reduction reactions.

In the presence of transition metals such as iron and copper, H_2_O_2_ can form non-selective reactive and toxic hydroxyl radicals (HO·) via the Fenton reaction [133,135,136,137]. Studies show that H_2_O_2_ is a particularly intriguing candidate as an intracellular and intercellular signaling molecule because it is neutral and passes through the membrane [138,139,140,141,142]. Specifically, H_2_O_2_ can oxidize thiol (-SH) cysteine residues and form sulfonic acid (–SOH), which can be glutathionylated (–SSG), forming a disulfide bond (–SS–) with neighbouring thiols, or can form sulfenyl amide (–SN–) with amides. Some of the well-known antioxidant flavonoids (quercetin, rutin) have been reported to act as prooxidants in the presence of transition metals [143]. The prooxidative activity of copper-initiated flavonoids also depends on the number of free OH groups in their structure [19,144], as well as their arrangement on the flavonoid skeleton. The more OH groups a flavonoid has, the stronger its prooxidant activity. In addition, flavonoids with catecholic OH groups on the B ring have been shown to have a lower bond dissociation enthalpy, with the OH group being the most active [19,33], especially when the catecholic OH groups are in the 3′ and 4′ or 4′ and 5′ positions (Figure 1). Methylation and possibly other O-modifications of flavonoid OH substitutions inactivate both antioxidative and prooxidative actions of flavonoids. The antioxidant activity of quercetin was found to be higher in aglycone than in aglycone [19,78]. A similar conclusion holds for flavonols in general [19] regarding both their antioxidative and prooxidative activities [134]. In the aglycone form, quercetin is more lipophilic and may interact with the polar heads of phospholipid bilayers by locating near the surface of the membrane [145].

The study shows to some extent that grape seed extract, which is considered an antioxidant dietary supplement, may also have prooxidant effects, depending on the dose, duration of intake, and other dietary components [146]. Low molecular weight polyphenols have higher antioxidant and prooxidant activity levels, although if certain conditions are met (e.g., high pH, high concentrations of transition metal ions, presence of oxygen molecules) they also have higher prooxidant activity [147,148]. Therefore, it is necessary to consider the dual behaviour of polyphenols and the accompanying environmental factors, such as the presence of transition metals in feed residues or the pH of the diet when developing a dietary plan for ruminants in order to avoid the side effects of the possible prooxidant activity of polyphenols in ruminant diets.

## 9. Conclusions

The sustainability and profitability of livestock production are closely related to the breeding of domestic animals. This review paper presents current data on the dependence of ruminant diets in order to help make valid decisions about whether polyphenols have a positive or negative effect on reproduction. Successful fertilization is influenced by various factors. One of the more important factors is the diet of ruminants, which today is (mostly) of plant origin. Plants synthesize secondary metabolites for their protection, which are polyphenols. Previous research has shown that the effects of polyphenols on the reproductive organs of animals can be both positive and negative. Polyphenols are known to have antioxidant, antimutagenic, and anti-inflammatory effects. One of the most important properties of polyphenols is their antioxidant activity. Oxygen and nitrogen free radicals are formed in every cell during metabolism. ROS and RNS have a dual role in biological systems, as they can be both harmful and beneficial to the cells. Low concentrations have a beneficial effect on cellular responses, such as defense against infectious agents, as well as in the maintenance and transmission of numerous cellular signals. High concentrations damage molecules in the cells, including lipids and membranes, proteins, and nucleic acids (so-called oxidative stress). Oxidative stress can also be viewed as an imbalance between prooxidants and antioxidants in the body. The prooxidative activity of copper-initiated flavonoids depends on the number of free OH groups in their structure.

Phytochemicals have been tested as feed additives due to their potential use as antioxidants, antimicrobials, immune stimulators, or modulators of rumen fermentation to generally improve metabolism and reduce the use of antibiotics. These products can affect animals by modulating appetite or digestive functions and processes (e.g., fiber digestibility, volatile fatty acid production levels in the rumen), interacting with immune, endocrine, or metabolic systems and increasing their efficiency (milk yield and composition). Feeding cows with a strain that has a high concentration of phytosterols can disrupt the estrous cycle and can cause certain ovarian dysfunctions during early pregnancy. The effects of phytoestrogenic clover isoflavones on the hormonal balance and pregnancy of heifers were confirmed via the use of different concentrations of isoflavone aglycones (genistein, daidzein, biochanin A, and formononetin), where higher concentrations led to reduced fertility in heifers.

Catechol polyphenols have a preventive effect on infertility by protecting germ cells and eggs from oxidative damage, as shown in numerous studies with EGCG. Protection against ROS in reproductive organs is achieved using non-enzymatic antioxidants, which are generally from the class of polyphenols and antioxidant enzymes. Polyphenols in low concentrations have been shown to reach fertilization cells and neutralize free radicals. It has been confirmed that metabolites of isoflavones (genistein, daidzein, biochanin A, and formononetin) can lead to hormonal imbalance and reduced fertility in heifers. Metabolites (equal and p-ethyl-phenol) prevent the formation of bovine corpus luteum and inhibit the synthesis of progesterone P4, which prepares the uterus for ovum implementation and pregnancy. From the above, it is concluded that the choice of ruminant food should be approached with caution, and that it is desirable to determine the total polyphenols and phytosterols. The available experimental results clearly indicate that the concentration of polyphenols and the diversity of substances contained in the diet (convective or grazing) can positively or negatively affect reproduction.

## Abbreviations

ART	assisted reproductive techniques
ATP	adenosine triphosphate
CAT	catalase
CYP	cytochrome P-450-dependent mixed function oxidase
E2	estradiol
EC-SOD (SOD3)	superoxide dismutase 3
EGCG	epigallocatechin-3-gallate
ERα	estrogen receptor alpha
ERβ	estrogen receptor beta
GPx	glutathione peroxidase
GSH	glutathione
GSSG	oxidized glutathione
GST	glutathione transferase
GTEx	green tea extract
LH	luteinizing hormone
IGF-1	insulin-like growth factor 1
NO	nitric oxide
OxSt	oxidative stress
PGE	prostaglandin E2
RNS	reactive nitrogen species
ROS	reactive oxygen species
SOD 1	superoxide dismutase 1
SOD 2	superoxide dismutase 2
T3	thyroid hormones 3
T4	thyroid hormones 4
VFA	volatile fatty acid
